# Prevalence of hepatitis C in a Swiss sample of men who have sex with men: whom to screen for HCV infection?

**DOI:** 10.1186/1471-2458-14-3

**Published:** 2014-01-06

**Authors:** Axel J Schmidt, Luis Falcato, Benedikt Zahno, Andrea Burri, Stephan Regenass, Beat Müllhaupt, Philip Bruggmann

**Affiliations:** 1 Checkpoint Zurich, Konradstrasse 1, 8005 Zurich, Switzerland; 2 Sigma Research, London School of Hygiene and Tropical Medicine, London, UK; 3 Arud Centers for Addiction Medicine, Konradstrasse 32, 8005 Zurich, Switzerland; 4 Institute of Psychology, University of Zurich, Binzmühlestrasse 14, 8050 Zurich, Switzerland; 5 Clinic of Immunology, University Hospital Zurich, Häldeliweg 4, 8044 Zurich, Switzerland; 6 Division of Gastroenterology and Hepatology, University Hospital Zurich, Rämistrasse 100, 8091 Zurich, Switzerland

**Keywords:** HCV, Hepatitis C prevalence, MSM, Switzerland, NIDU

## Abstract

**Background:**

While the numbers of hepatitis-C-virus (HCV) infections among men who have sex with men (MSM) who are co-infected with the human immunodeficiency virus (HIV) are on the rise, with vast evidence for sexual transmission of HCV in this population, concerns have also been raised regarding sexual HCV-transmission among MSM without HIV infection. Therefore, the aim of this study was to estimate the prevalence of hepatitis C among MSM without HIV diagnosis in Zurich (Switzerland).

**Methods:**

Participants were recruited from a gay health centre and various locations such as dark rooms, saunas and cruising areas in Zurich. Participants self-completed a questionnaire assessing known and suspected risk factors for HCV-infection, and provided a blood sample for detection of past (antibodies) and present (core antigen, RNA) infections with HCV.

**Results:**

In total, 840 MSM aged 17-79 (median: 33 years) underwent HCV-testing and completed the questionnaire, among whom 19 reported living with HIV. Overall, seven tested positive for HCV-antibodies, and two were also positive for HCV core antigen and HCV-RNA–these two were immigrants, one from a country where HCV is endemic. None of the seven were aware of their infection. The seroprevalence of hepatitis C among the 821 non-HIV-diagnosed MSM was 0.37% (95%-CI: 0.12-1.69%), and one man harboured replicating virus (0.12%; 0.02-0.69%), resulting in a number needed to test of 821 to detect one active infection. Significant univariable associations of lifetime HCV-infection were found with known HIV-diagnosis (OR=72.7), being tattooed (OR=10.4), non-injection use of cocaine/amphetamines (OR=8.8), and non-Swiss origin (OR=8.5). For MSM without HIV-diagnosis, the only variable marginally associated with positive HCV-serostatus was being tattooed (OR=8.3). No significant associations were observed with reported injection drug use, unprotected anal intercourse, sexual practices that may lead to mucosal trauma, or proxy measures for group sex and lesion-prone STIs.

**Conclusions:**

Our findings suggest that in Switzerland, hepatitis C among MSM without diagnosed HIV is not more prevalent than in the general population. We found no evidence of elevated rates of sexual transmission of HCV among MSM without HIV-infection. Therefore, we currently see no reason for promoting HCV-testing among all MSM in Switzerland.

## Background

In Switzerland, a yearly average of 1500 new diagnoses of active hepatitis C virus (HCV) infections (positive for HCV core antigen and PCR) was reported for the years 2010-2012 (18.7/100000 inhabitants) [1]. The number of people in Switzerland living with HCV is difficult to estimate; however the proportion exposed to the virus (anti-HCV prevalence) has been estimated to be 0.7%-1.0%, based on testing blood donors and pregnant women [2]. Models that take the non-representativeness of these sub-populations into account have suggested that the true anti-HCV prevalence in the Swiss general population might be between 1.25% and 1.75% [3]. 75% of individuals infected with HCV develop chronic infection, which is linked to the development of hepatic fibrosis, cirrhosis and hepatocellular carcinoma [4].

Percutaneous exposures to blood, such as blood transfusion, injection drug use (IDU), unsterile tattooing and body piercings are well-established primary sources of HCV infection [5,6], and there is increasing evidence that non-injection drug use (NIDU) such as intranasal cocaine is an independent risk factor for hepatitis C. There is vast evidence that hepatitis C is extremely rarely transmitted via sexual contact among HCV-serodiscordant heterosexual couples [7-11]. However, in the last decade, outbreaks of HCV-infection have been documented among HIV-positive men who have sex with men (MSM) [12-22]. Data from the CASCADE study, including HIV-infected MSM from eleven HIV seroconverter cohorts in eight European countries and one such cohort in Canada [23], showed that HCV incidence increased 4-to 6-fold between 1995 and 2007. Also, a recent Swiss HIV Cohort Study (SHCS) publication has demonstrated that HCV incidence increased 18-fold among HIV-positive MSM between 1998 and 2011 [24].

The exact modes of non-IDU related HCV transmission among HIV positive MSM are still a matter of debate. While several studies have identified group sex, traumatizing sexual practices, and receptive fisting as risk factors for hepatitis C, and while there is little doubt that sexual practices involving blood contact can facilitate transmission of a blood-borne virus such as HCV [13,18,25], the role of contaminated seminal fluid for HCV transmission is still debated [6,25-29]. Lately, a few cases of possibly sexually acquired HCV infection among MSM without HIV infection have been reported in the Netherlands [30], and concerns have been raised that the HCV epidemic among HIV-positive MSM could expand to MSM without HIV infection [31]. MSM as such are often considered to be at higher risk for HCV compared to the heterosexual general population, and some authors have recommended routine screening of MSM in Switzerland [32,33].

Very little is known about the prevalence of HCV in non-HIV-positive MSM living in Switzerland, as there is a dearth of systematic epidemiologic studies. Notification forms in Switzerland include the gender of sexual partners, but no MSM-specific epidemiological data have been published. Such analyses from Germany have shown that HCV notifications for MSM have been increasing over the last decade; however, as is the case in Swiss surveillance, data on HIV serostatus are not routinely collected as part of German hepatitis C notification [34]. Therefore the aim of the present study was to narrow this gap by estimating the prevalence of current and past HCV infection among non-HIV-diagnosed MSM in Switzerland and–if applicable–to identify potential risk factors for HCV infection.

## Methods

### Recruitment

*Checkpoint Zurich* is a gay health project run by gay men that started in 2006. Clients can test for HIV and syphilis (using rapid point of care tests) and for asymptomatic bacterial sexually transmitted infections (STIs) such as pharyngeal and rectal gonorrhoea or chlamydiosis. *Checkpoint Zurich* employs medical staff for hepatitis A and B vaccinations, clinical examinations, medical counselling, post-exposure prophylaxis to prevent HIV infection, and medical treatment for HIV and other STIs. In 2007, *Checkpoint Zurich* initiated new outreach strategies (“*Checkpoint Mobile*”) across various locations (dark rooms, saunas, motorway rest stops, and private sex parties), offering on-site testing for HIV and/or syphilis [35]. Overall, both services demonstrated high levels of acceptance and demand in the Swiss MSM community.

From January 2009 to July 2010, MSM clients visiting *Checkpoint Zurich* to test for HIV and/or syphilis, and MSM approached through *Checkpoint Mobile* were asked to participate in the present study. After obtaining detailed study information by specially trained male gay nurses or fieldworkers, and after providing written consent to be tested for these infections, participants self-completed an anonymous paper and pencil questionnaire covering detailed sexual practices, other potential exposures for HCV infection such as injection drug use (IDU) or non-injection drug use (NIDU) of cocaine/amphetamines, tattoos, piercings, previous blood transfusions, and previous diagnosis of HIV or other STIs. All men provided a venous blood sample, to be used for syphilis and HIV rapid tests (at *Checkpoint Zurich*) and subsequently sent to the University Hospital of Zurich for HCV screening. While HCV-testing was free of charge, 30 Swiss Francs was charged for the combined HIV and syphilis test.

Participants were given a printed single identifier to use when calling *Checkpoint Zurich* to receive their test results for hepatitis C (or, in the case of *Checkpoint Mobile* clients, to use when receiving personal post-test HIV counselling at *Checkpoint Zurich*). In case of positive test results for any of the three infections, participants were offered further assistance, information and psychological support and treatment at *Checkpoint Zurich*, if indicated.

### Serology

HCV-screening was performed at the Clinic of Immunology, University Hospital of Zurich, by parallel testing for antibodies against HCV and HCV core antigen (*ARCHITECT® Anti-HCV*; and *ARCHITECT® HCV Ag;* Abbott*,* Wiesbaden, Germany). All specimens that were reactive or borderline reactive for either antigen or antibody were confirmed by quantitative HCV PCR (*COBAS® AmpliPrep*/ *COBAS® TaqMan® HCV, v2.0*, Roche Basel, Switzerland). HIV and Syphilis rapid test results were not entered into the database and are thus not reported in this analysis.

### Self-reported measures

As on-site recruitment required efficient data collection, questions were kept simple, mostly with binary response options.

HIV status was queried as “positive”, “negative”, “unknown” and “I don’t want to say”. For their lifetime history of STI diagnoses, participants could indicate whether they had had syphilis, urethral gonorrhoea, rectal gonorrhoea, pharyngeal gonorrhoea, chlamydial infection, genital herpes, genital or anal warts, hepatitis A, hepatitis B, hepatitis C, and “others”. Where appropriate, colloquialisms for STIs were used.

Drug use was queried as “Do you use injection drugs?”, “Do you take cocaine/speed?”, “… ecstasy”, “… cannabis”, “… poppers”. As for other known exposure risks for HCV infection, participants could indicate if they had had a blood transfusion prior to 1985, been tattooed, pierced, had their penis pierced, and whether tattooing or piercing was performed in Switzerland or abroad.

Unprotected anal intercourse (UAI) was queried as “Do you practice unprotected anal intercourse?”, and answer options (Yes/No) referred to “with my steady partner”, “with known casual partners”, and “with anonymous partners”.

To address bleeding associated with sexual intercourse, a filter question was included asking whether participants engaged in “hard” sexual practices that may lead to bleeding. This filter question was followed by a list of various sexual behaviours, to be checked as active and/or passive, including fisting, whipping, genital torture, dildo usage and anal dilatation, enema and catheterisation. It was further asked where and with what type of sexual partner “hard” sexual practices were performed.

### Constructed measures

Participants’ countries of origin were grouped according to the latest published data on prevalence of HCV-antibodies in the general population: <1.5%; 1.5-3.5% (including Switzerland); >3.5% [36].

To construct a proxy measure for STIs that have been associated with HCV transmission in other studies [29] we combined lifetime history of syphilis, rectal gonorrhoea, and chlamydial infection, and labelled this proxy measure “lesion-prone STIs”.

We constructed a proxy measure for “group sex” by combining (a) reports of 10 or more sexual partners in the past 12 months with (b) reports of engaging in “hard” practices (see above) in sex clubs or at private sex parties.

### Statistical analyses

Except for age and the number of sexual and UAI partners in the last 12 months, all variables were either originally binary or later dichotomized. Fisher’s exact test was used to compare groups of men with and without HCV-antibodies, as the number of men with HCV was below 20. For all analyses, a *p* value less than 0.05 or odds ratios (OR) with a 95% confidence interval (CI) not including “1” were considered statistically significant. Under the assumption that HCV seroprevalence in this sample was 1% and that 10% of HCV negative men had been exposed to HCV transmission, power calculations showed the study would have a power of 80% to detect an OR of 6.0 for the association of HCV seropositivity with risky behaviour. Power calculations were performed with G*Power 3.1 [37]. Confidence intervals of proportions were calculated according to *Newcombe et al*. 1998 [38]. All other analyses were performed using IBM® SPSS® Statistics, Version 20.

### Ethical approval

The Ethics Commission of the Canton of Zurich, Switzerland, approved the study (EK-1715); and further approval for *Checkpoint Mobile* recruitment was obtained from the owners and operators of the targeted locations.

## Results

### Study participants

Out of 884 men approached, 845 (95.6%) provided a blood sample and informed consent regarding HCV-testing, and usage of their anonymous data for scientific purposes. Five questionnaires were excluded due to missing values in 5 or more key variables; the analytic sample thus consisted of *n* = 840 study participants (95.0%).

No information was systematically collected as to whether clients were recruited at *Checkpoint Zurich* or via *Checkpoint Mobile*; however, field workers estimate that the latter accounted for one quarter of participants.

Nineteen individuals (2.3% of the analytic sample) reported a diagnosis of HIV; and of the remaining 821 men without known HIV infection, 579 (70.5%) were self-reportedly HIV-negative, 188 (22.9%) said they did not know their current HIV status, and 54 (6.6%) did not want to disclose it. Socio-demographic and other health and behaviour-related characteristics of study participants are shown in Table 1, both for the total sample and separately for men with and without HIV diagnosis. Participants’ age ranged from 17 to 79 years with a median of 33 years. 178 (21.2%) had a country of origin other than Switzerland; and 9 (1.1%) originated from countries where HCV is endemic (>3.5%–including four men from China and three from Turkey).

**Table 1:** Hepatitis C prevalence, socio-demographic, behavioural, and sexual health related sample characteristics, stratified by HIV diagnosis, in a sample of men who have sex with men from Zurich

**Characteristic**	**Total**			**No known HIV infection**			**HIV diagnosed**		
	***n*** **= 840**			***n*** **= 821**			***n*** **= 19**		
**Hepatitis C prevalence**	**%**	**(95%-CI)**	***n***	**%**	**(95%-CI)**	***n***	**%**	**(95%-CI)**	***n***
Antibody*	0.8	(0.4-1.7)	7	0.4	(0.12-1.1)	3	21.1	(8.5-43.3)	4
Antigen*	0.2	(0.1-0.9)	2	0.1	(0.02-0.7)	1	5.3	(0.9-24.6)	1
**Age**	**Years**			**Years**			**Years**		
Range	17-79			17-79			19-59		
25%-, 50%-, 75%-quartiles	26, 33, 41			26, 33, 41			33, 38, 47		
**Country of origin**	**%**	**(95%-CI**^**1**^**)**	***n***	**%**	**(95%-CI)**	***n***	**%**	**(95%-CI)**	***n***
By nationality									
Swiss	70.4	(67.2-73.3)	591	70.6	(67.4-73.6)	580	57.9	(36.3-76.9)	11
Non-Swiss	21.2	(18.6-24.1)	178	21.0	(18.3-23.9)	172	31.6	(15.4-54.0)	6
Unknown	8.5	(6.8-10.5)	71	8.4	(6.7-10.5)	69	10.5	(2.9-31.4)	2
By national HCV prevalence^2^									
< 1.5%	3.5	(2.4-4.9)	29	3.3	(2.3-4.7)	27	10.5	(2.9-31.4)	2
1.5-3.5% (incl. Switzerland)	87.0	(84.6-89.1)	731	87.3	(84.9-89.4)	717	73.7	(51.2-88.2)	14
> 3.5%	1.1	(0.6-2.0)	9	1.0	(0.5-1.9)	8	5.3	(0.9-24.6)	1
Unknown	8.5	(6.8-10.5)	71	8.4	(6.7-10.5)	69	10.5	(2.9-31.4)	2
**Blood exposures**	**Valid%**	**(95%-CI)**	***n***	**Valid%**	**(95%-CI)**	***n***	**Valid%**	**(95%-CI)**	***n***
Blood transfusion before 1985	1.3	(0.7-2.4)	11	1.4	(0.8-2.4)	11	0.0	(0.0-16.8)	0
Piercing	24.7	(21.8-27.8)	198	24.5	(21.6-27.7)	192	31.6	(15.4-54.0)	6
Performed abroad	6.1	(4.6-7.9)	51	6.0	(4.5-7.8)	49	10.5	(2.9-31.4)	2
Genital piercing	2.8	(1.8-4.2)	22	2.7	(1.8-4.1)	21	6.2	(1.1-28.3)	1
Tattoo	19.9	(17.2-23.0)	145	19.6	(16.8-22.7)	139	35.3	(17.3-58.7)	6
Performed abroad	7.9	(6.2-9.9)	66	7.7	(6.0-9.7)	63	15.8	(5.5-37.6)	3
**Current drug use**	**Valid%**	**(95%-CI)**	***n***	**Valid%**	**(95%-CI)**	***n***	**Valid%**	**(95%-CI)**	***n***
IDU^3^	3.3	(2.2-5.0)	22	3.1	(2.0-4.7)	20	13.3	(3.7-37.9)	2
NIDU of cocaine / amph.^4^*	13.6	(11.3-16.3)	99	12.8	(10.6-15.5)	91	47.0	(26.2-69.0)	8
Ecstasy*	12.2	(10.0-14.8)	87	11.8	(9.6-14.4)	82	31.3	(14.2-55.6)	5
Cannabis*	20.4	(17.6-23.4)	151	19.8	(17.1-22.9)	144	46.7	(24.8-69.9)	7
Any of the above*	50.0	(46.6-53.4)	412	48.9	(45.5-52.4)	394	94.7	(75.4-99.1)	18
**(Continued)**	**Total**			**No known HIV infection**			**HIV diagnosed**		
	***n*** **= 840**			***n*** **= 821**			**n = 19**		
**Sexual partners last year**	**Number**			**Number**			**Number**		
Range	0-500			0-500			0-200		
25%-, 50%-, 75%-quartiles	4, 6, 15			4, 6, 12			10, 20, 50		
	**Valid%**	**(95%-CI)**	***n***	**Valid%**	**(95%-CI)**	***n***	**Valid%**	**(95%-CI)**	***n***
More than 10 sexual partners*	30.1	(27.1-33.3)	250	29.2	(26.2-32.4)	237	68.4	(46.0-84.6)	13
**UAI**^5^**partners last year**	**Number**			**Number**			**Number**		
Range	0-200			0-60			0-200		
25%-, 50%-, 75%-quartiles	0, 1, 1			0, 1, 1			0, 1, 6		
	**Valid%**	**(95%-CI)**	***n***	**Valid%**	**(95%-CI)**	***n***	**Valid%**	**(95%-CI)**	***n***
More than 10 UAI partners*	1.1	(0.6-2.0)	9	0.6	(0.3-1.4)	5	21.1	(8.5-43.3)	4
**UAI episodes last year**	**Valid%**	**(95%-CI)**	***n***	**Valid%**	**(95%-CI)**	***n***	**Valid%**	**(95%-CI)**	***n***
With steady partner*	48.5	(45.1-52.0)	384	49.1	(45.6-52.6)	380	23.5	(9.6-47.2)	4
With known casual partners	25.1	(22.2-28.2)	201	24.9	(22.0-28.1)	195	33.3	(16.3-56.3)	6
With anonymous partners*	11.8	(9.7-14.3)	92	11.3	(9.3-13.8)	86	31.6	(15.4-54.0)	6
“Hard” sexual practices*^6^									
Any that might lead to bleeding	12.1	(10.1-14.5)	102	11.7	(9.7-14.1)	96	31.6	(15.4-54.0)	6
Fisting, receptive*	4.0	(2.9-5.6)	34	3.8	(2.7-5.3)	31	15.8	(5.5-37.6)	3
Dildo usage, receptive	6.9	(5.4-8.8)	58	6.7	(5.2-8.6)	55	15.8	(5.5-37.6)	3
Anal dilatation, receptive	2.3	(1.5-3.5)	19	2.1	(1.3-3.3)	17	10.5	(2.9-31.4)	2
Enema, receptive	1.2	(0.6-2.2)	10	1.1	(0.6-2.1)	9	5.3	(0.9-24.7)	1
Catheterisation, receptive	1.2	(0.6-2.2)	10	1.2	(0.6-2.2)	10	0.0	(0.0-16.8)	0
With steady partner	6.9	(5.3-8.8)	57	6.7	(5.2-8.6)	54	15.8	(5.5-37.6)	3
With known casual partners	11.3	(9.3-13.6)	94	10.9	(8.9-13.2)	89	26.3	(11.8-48.8)	5
With anonymous partners*	5.9	(4.5-7.7)	49	5.5	(4.2-7.3)	45	21.1	(8.5-43.3)	4
At home	12.6	(10.6-15.1)	105	12.3	(10.2-14.7)	100	26.4	(11.9-48.9)	5
At private sex parties*	4.6	(3.4-6.3)	38	4.2	(3.0-5.8)	34	21.1	(8.5-43.4)	4
In sex clubs*	6.9	(5.4-8.8)	57	6.3	(4.8-8.2)	51	31.6	(15.4-54.0)	6
In cruising areas*	3.4	(2.4-4.9)	28	3.1	(2.1-4.5)	25	15.8	(5.5-37.6)	3
Group sex^7^*	5.4	(4.0-7.1)	45	4.9	(3.6-6.6)	40	26.3	(11.8-48.8)	5
**Lifetime history of STIs**^8^	**Valid%**	**(95%-CI)**	***n***	**Valid%**	**(95%-CI)**	***n***	**Valid%**	**(95%-CI)**	***n***
Urethral gonorrhea*	20.2	(17.6-23.1)	168	19.6	(17.0-22.5)	159	47.4	(27.4-68.3)	9
Rectal gonorrhea	1.7	(1.0-2.8)	14	1.6	(0.9-2.7)	13	5.3	(0.9-24.7)	1
Pharyngeal gonorrhea	1.5	(0.9-2.6)	13	1.6	(0.9-2.7)	13	0.0	(0.0-16.8)	0
Chlamydial infection*	10.9	(9.0-13.2)	91	10.5	(8.6-12.8)	85	31.6	(15.4-54.0)	6
Syphilis*	8.1	(6.4-10.1)	68	7.1	(5.5-9.1)	58	52.6	(31.7-72.6)	10
Hepatitis A	2.2	(1.4-3.4)	18	2.0	(1.2-3.2)	16	10.5	(2.9-31.4)	2
Hepatitis B*	2.3	(1.5-3.5)	19	1.8	(1.1-3.0)	15	21.1	(8.5-43.4)	4
Genital herpes	2.5	(1.6-3.8)	21	2.4	(1.6-3.7)	20	5.3	(0.9-24.7)	1
Genital or anal warts*	7.8	(6.2-9.8)	65	7.2	(5.6-9.2)	59	31.6	(15.4-54.0)	6
Lesion-prone STIs^9^*	18.2	(15.8-21.0)	153	17.2	(14.7-19.9)	141	63.2	(41.1-80.9)	12

*Fisher’s exact test <0.05; ^1^CI: confidence interval; ^2^National HCV prevalence according to *Modh Hanafiah et al*. 2012 [36]; ^3^IDU: injection drug use; ^4^NIDU of cocaine/amph.: non-injection drug use of cocaine or amphetamines; ^5^UAI: unprotected anal intercourse; ^6^“Hard” sexual practices that may lead to bleeding; ^7^group sex: defined as reporting 10 or more sexual partners in the past 12 months and engaging in “hard” practices (see above) in sex clubs or at private sex parties; ^8^STIs: sexually transmitted infections; ^9^Lesion-prone STIs: syphilis, rectal gonorrhoea, or chlamydial infection.

Blood transfusions prior to 1985 were reported by 11 men (1.3%) and solely by individuals born before 1980, underlining the plausibility of the answers given. The question on IDU garnered the highest number of missing responses (*n* = 176; 21%); however this missingness was equally distributed across HIV diagnosis and HCV infection, respectively.

Except for blood transfusion and piercing, all suspected HCV-related risk factors such as IDU, NIDU (particularly consumption of cocaine/amphetamines), receptive fisting, UAI with anonymous partners, as well as proxy measures for group sex and lesion-prone STIs were more pronounced among HIV-diagnosed MSM (Table 1).

### Prevalence of hepatitis C and number needed to test

Evidence for acute or chronic infection with HCV was found in 2 individuals (positive for HCV antibodies, HCV antigen and HCV PCR), and 5 individuals had cleared their infection (positive for HCV antibodies but negative for HCV antigen and HCV PCR), resulting in an overall anti-HCV prevalence of 0.83% (*n* = 7; 95%-CI: 0.40-1.71%). None of the 7 individuals with HCV antibodies reported a diagnosis of hepatitis C in the past–suggesting they were unaware of their (past) infection, and suggesting that clearance of HCV infection was spontaneous and not due to medical treatment.

Among MSM not diagnosed with HIV, one individual showed evidence of active (acute or chronic) HCV infection, and two had spontaneously cleared their infection, resulting in an anti-HCV prevalence of 0.37% (*n* = 3; 95-CI: 0.12-1.07%). The number of non-HIV-diagnosed MSM *Checkpoint* clients needed to test in order to detect one active HCV infection (NNT) was 821 (95%-CI: 146-4545).

**Figure 1:**
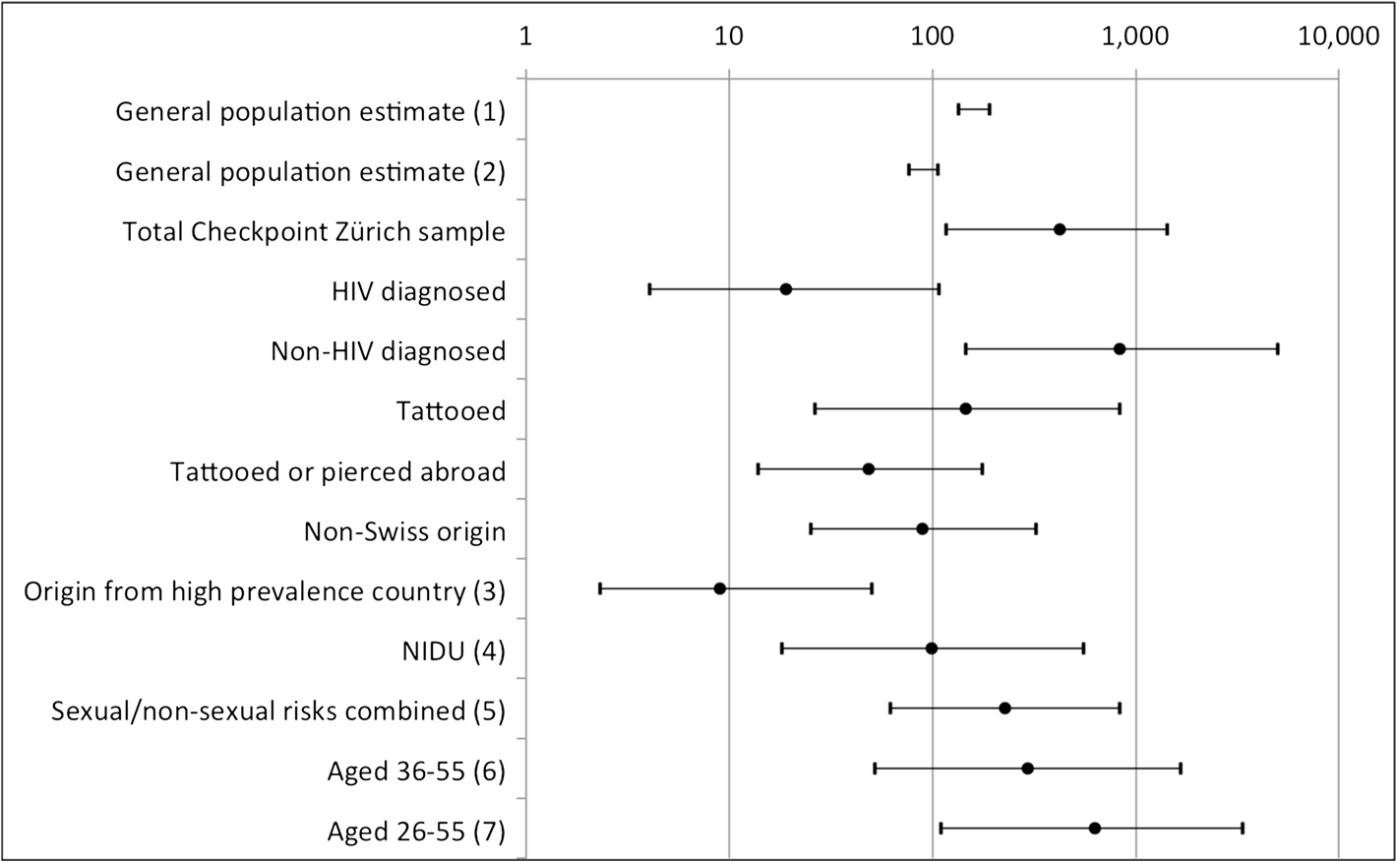
**Number needed to test (NNT) to detect one active HCV infection Number needed to test (on a logarithmic scale) to detect one active HCV infection, in different scenarios for targeted HCV-testing.** The two intervals at the top are based prevalence estimates for the Swiss general population, assuming that 75% of those ever infected develop chronic infection, and each reflecting the respective lower and upper limit. The remaining eleven point estimates with 95% confidence intervals represent MSM recruited at *Checkpoint Zurich*, reflecting the whole sample (*n* = 840; thus including 19 men with diagnosed HIV) or sub-samples based on individual characteristics. (1) Estimate based on blood donors and pregnant women [2]; (2) Estimate based on mathematical modelling [3]; (3) High anti-HCV prevalence (>3.5%) countries were defined according to *Modh Hanafiah et al*. 2012 [36]; (4) Non-injection drug use (NIDU) of cocaine/amphetamines; (5) Any of the following: NIDU of cocaine/amphetamines, being tattooed, being pierced, receptive fisting, group sex (proxy measure), or a history of lesion-prone STIs (proxy measure); (6-7) Birth cohort screening (1955-1974/1955-1984) as discussed as add-on strategies by *Bruggmann et al. 2013*[39].

Among all participants, 450 (53.6%) reported at least one of the following: NIDU of cocaine/amphetamines, being tattooed, being pierced, receptive fisting, group sex (proxy measure), or a history of lesion-prone STIs. If only this sub-group were to be screened for HCV, the NNT would be reduced to 225 individuals (95%-CI: 62-820). However with HIV-diagnosed MSM excluded from this sub-group, the NNT would be 433 (77-2439). Figure 1 shows the NNT in various scenarios for targeted HCV-testing, based on the analytic sample.

All three anti-HCV positive individuals without known HIV infection–aged 24, 28, and 60 years–denied both IDU (no missing responses, see above) and NIDU, reported no history of blood transfusion prior to 1985, fisting, group sex (proxy measure), lesion-prone STIs, or engaging in “hard” sexual practices that may lead to bleeding. The two younger men reported being tattooed, and the individual with the active HCV infection had been tattooed abroad–possibly in the Czech Republic, his country of origin. (The HCV prevalence in the Czech Republic is estimated to be 1.5%-2.0% [2]).

### Factors associated with lifetime HCV-prevalence

In a univariable analysis (Table 2; *n* = 840), the following factors were significantly associated with anti-HCV seropositivity: being diagnosed with HIV (OR = 72.7), being tattooed (OR = 10.4), NIDU of cocaine/amphetamines (OR = 8.8), and non-Swiss origin (OR = 8.5). None of the four individuals co-infected with HIV and HCV (0.5% or the analytic sample) was of Swiss origin, but came from Brazil, China, Spain, and Italy, countries where HCV is more prevalent than in Switzerland [2,36].

**Table 2:** Exposure risks with significant associations comparing HCV antibody positive and negative men who have sex with men from Zurich, stratified by HIV diagnosis

**Factor (exposure risk)**		**HCV pos.**^**1**^		**HCV neg.**		**Fisher’s**^**2**^	**OR**^**3**^	**95% CI**^**4 **^**of OR**
**(Without substitution of missing values)**		***n***	**%**	***n***	**%**			
**HIV**^5^**diagnosis**								
Total sample (*n* = 840)	Yes	4	21.1	15	78.9	0.000	72.70	14.95-353.56
	No	3	0.4	818	99.6			
**Non-Swiss origin**								
Total sample (valid *n* = 769)	Yes	5	2.8	173	97.2	0.009	8.51	1.64-18.75
	No	2	0.3	589	99.7			
No known HIV infection (valid *n* = 752)	Yes	1	0.6	171	99.4	0.542	1.69	0.15-18.75
	No	2	0.3	578	99.7			
HIV diagnosed (valid *n* = 17)	Yes	4	66.7	2	33.3	0.006	*n.a.*^7^	
	No	0	0.0	11	100.0			
**Tattooed**								
Total sample (valid *n* = 727)	Yes	5	3.4	140	96.6	0.004	10.36	1.99-53.94
	No	2	0.3	580	99.7			
No known HIV infection (valid *n* = 710)	Yes	2	1.4	137	98.6	0.100	8.32	0.75-92.43
	No	1	0.2	570	99.8			
HIV diagnosed (valid *n* = 17)	Yes	3	50.0	3	50.0	0.099	10.00	0.74-135.33
	No	1	9.1	10	90.9			
**NIDU**^6^**of cocaine or amphetamines NIDU only**								
Total sample (valid *n* = 727)	Yes	4	4.0	95	96.5	0.012	8.77	1.93-39.81
	No	3	0.5	625	99.5			
No known HIV infection (valid *n* = 710)	Yes	0	0.0	91	100.0	0.621	*n.a.*	
	No	3	0.5	616	99.5			
HIV diagnosed (valid *n* = 17)	Yes	4	50.0	4	50.0	0.053	*n.a.*	
	No	0	0.0	9	100.0			

^1^HCV pos./neg.: positive/negative for antibodies against hepatitis C virus; ^2^Fisher’s exact test; ^3^OR: odds ratio; ^4^CI: confidence interval; ^5^HIV: human immunodeficiency virus; ^6^NIDU: non-injection drug use; ^7^*n.a.*: not applicable (division by zero).

In the univariable analysis among non-HIV-diagnosed MSM (Table 2), being tattooed was marginally associated with HCV infection (OR = 8.3).

No significant associations were found between HCV-antibody positivity and age, past blood transfusions, being pierced or genitally pierced, IDU, number of sexual partners, number of UAI partners, UAI episodes, fisting, other “hard” sexual practices that may lead to bleeding, group sex (proxy measure), syphilis, or lesion-prone STIs.

## Discussion

### Prevalence of HCV and lack of evidence for sexual transmission among non-HIV-diagnosed MSM

In a sample of 821 non-HIV-diagnosed MSM recruited at a gay health centre in Zurich (Switzerland), we found an anti-HCV prevalence of 0.37% (0.12-1.69%); and a prevalence of active (acute or chronic) HCV infection of 0.12% (0.02-0.69%). To our knowledge, this is the first study to look at the prevalence of hepatitis C among non-HIV-diagnosed MSM in Switzerland. Although the level of reported sexually risky behaviours (UAI with different types of partners, numbers of sexual partners) and related indicators such as lifetime history of gonorrhoea and syphilis was substantial and comparable with other surveys on MSM in Switzerland [40], we found no evidence for elevated rates of sexual transmission of HCV in this population. Instead, being tattooed was marginally associated with a positive HCV serostatus, even when controlling for potential confounding factors. However, questions have been raised as to whether such associations might be due to residual confounding, as for low HCV-endemic countries such as Switzerland strict hygiene guidelines for tattoo and piercing studios have been implemented [41].

Overall, our results correspond well with previously published prevalence estimates from Sydney (Health in Men study), where 7 out of 824 HIV-negative men aged 18-75 years (0.85%) tested positive for HCV antibodies [42]. As in the Australian study, we found no associations between HCV seropositivity and sexual practices that could have plausibly facilitated blood exposure, such as UAI and fisting. However, in a later longitudinal analysis, the Australian researchers found five HIV-negative men who seroconverted to HCV positivity (incidence of 0.11 per 100 person-years). Only one seroconverter reported IDU, four reported sexual contact with HIV-positive men, and two had an incident ulcerative sexually transmitted infection [43].

In the Montreal-based Omega cohort study of HIV-negative MSM, despite an anti-HCV baseline prevalence of 2.9%, only 1 seroconversion was identified (incidence of 0.038 per 100 person-years), and this infection could be attributed to IDU. The authors concluded that sexual transmission of HCV among non-HIV-diagnosed MSM appeared to be rare in 2001 [44]. Based on 1,699 non-IDU MSM recruited in public health clinics in Seattle, San Diego, and New York City, a low (1.5%) prevalence of anti-HCV did not “support routine HCV testing of all MSM” [45].

In a recent study conducted in London, the anti-HCV prevalence among 965 HIV-negative men was 1.2% (0.6-2.1%) and thus “higher, but not significantly higher, than that in the general population (0.67%)” [46]. Although the prevalence estimate among HIV-negative men in London was three times higher than in our Zurich sample, the authors recommend selective, not routine, HCV-testing among MSM according to the individual risk profile.

A recently published systematic review of studies on HCV-incidence among MSM concluded that the pooled incidence measure for HIV-negative MSM “is low and approximates that seen in the heterosexual population, where screening is not recommended” [47]. The authors further suggest validating “the use of factors, such as at-risk sexual behaviour and the serology of sexual partners which are not always considered in medical assessments for targeting routine HCV-screening in HIV-negative MSM populations”.

In the *Australian Trial in Acute Hepatitis C* (ATAHC), sexual transmission among HIV-uninfected individuals “was unusual but was almost always in the context of contact with a known HCV-infected partner” [48].

Multiple studies have investigated the associations between HCV and UAI to identify possible routes of HCV transmission, producing divergent results. Although significant associations between UAI and HCV infection in MSM have been reported, the validity of some of these studies [19] is limited by a lack of adjustment for confounding behavioural factors. A recent prospective longitudinal cohort study [24] found a significant association between UAI and HCV infection, but did not include potential confounders that could explain other sexual or non-sexual routes of HCV infection, such as frequency of anal intercourse, group sex, sex-associated bleeding, traumatising sexual practices such as fisting, use of PDE-inhibitors, lesion-prone STIs (i.e. sexual), or (procto-)surgical interventions [25], tattooing or NIDU (i.e. non-sexual).

We were not able to replicate the commonly reported association between IDU and HCV infection, as none of the 22 MSM in our sample reporting IDU tested positive for HCV antibodies. Given the low number and proportion (3.3%) of MSM with IDU in the analytic sample, our study was not sufficiently powered to detect IDU as a risk factor for hepatitis C. However, the prevalences we found for current IDU, 3.1% among non-HIV-diagnosed MSM (95%-CI: 2.0-4.7), and 13.3% among HIV-diagnosed MSM (3.7-37.9%), are very similar to results from the largest Swiss MSM sample [40], where the respective values (IDU in the last 12 months among MSM in Zurich) were 3.7% and 17.0%.

Existing epidemiological studies on the association between non-injection drug use (NIDU) and HCV produced fairly consistent findings, indicating elevated HCV prevalence (2.3%-17%) in different NIDU samples. However, it still remains unclear whether HCV in people with NIDU can be attributed to undisclosed IDU or other residual confounding [49-51], although the biologic plausibility of intranasal transmission has been demonstrated [52-55]. In our sample NIDU of cocaine/amphetamines had substantial interaction with known HIV infection; and although none of the HCV-infected MSM without HIV diagnosis reported NIDU of snorting drugs, we cannot rule out that this behaviour constitutes an independent risk factor for HCV infection. Again, these findings highlight the need for further research in this area with more rigorous methodology needed to disentangle the specific pathways and risk factors for transmission.

With respect to HCV transmission among MSM–sexual or non-sexual–little attention has been given to the influence of social and sexual networks [56-58]. Particularly for gay men living with HIV, such networking has been described as serosorting, a practice sometimes conceptualised as a strategy to reduce HIV transmission risk. However, a more important motive for HIV-positive MSM to establish sexual contacts preferably with HIV-seroconcordant partners is to reduce stigma and sexual rejection [59]. This consequently leads to a higher chance of exposure to STIs [60,61]. With respect to hepatitis C it has further been hypothesised that “assortative mixing, or partnership formation within subgroups, is common and that this mixing pattern might result in HCV being more prevalent” [62]. We hypothesize that sexual and social networks of MSM are the key to understanding the dynamics of sexual as well as non-sexual transmission of HCV. Taking into account networks and sub-group formation would in our view help to explain why HCV among HIV-positive MSM has spread so rapidly in the last decade [24]. This would also explain why the HCV epidemic among HIV-positive MSM has not jumped to MSM without HIV diagnosis, despite individual behaviour that would facilitate HCV transmission in other sub-populations or networks.

### Number needed to test and targeted HCV-testing

The number of non-HIV-diagnosed MSM *Checkpoint* clients needed to test in order to detect one active HCV infection (NNT) was 821 (95%-CI: 146-4545). Recent Swiss publications recommend HCV screening e.g. for persons who use or have used intranasal drugs, persons with body piercings or tattoos if performed in poor hygienic environment, and also for men who have sex with men [32]. Our study, like all other published studies on HIV-negative MSM, does not support the screening of MSM without HIV diagnosis for HCV antibodies, not even if restricted to those with NIDU of cocaine/amphetamines, tattoos, piercing, receptive fisting, group sex (proxy measure), or a history of lesion-prone STIs. The only sub-population where the NNT was significantly lower than for the general population (Figure 1) were individuals originating from high prevalence countries, individuals with HIV diagnosis (though not significant due to small numbers), and individuals who reported having been tattooed or pierced abroad. Other authors have suggested further evaluating birth cohort screening for HCV [39]. None of the suggested birth cohorts, when applied to our Zurich sample of MSM, resulted in a NNT lower than for the general population.

### Limitations

Several study limitations need to be considered: First, the cross-sectional study design precludes the calculation of incidence rates and the investigation of causation, as it is uncertain whether reported behaviour preceded HCV infection or not. Second, as information on HIV diagnosis was based on self-report, the true HIV prevalence among participants is likely to be underestimated. Unfortunately, as anonymous testing was part of our study design, the serological test results could not be added post-hoc. However, focusing on MSM without serologically confirmed HIV serostatus reflects much better the situation of health care providers and counsellors when deciding whom to offer HCV-screening in a clinical setting. Third, the study has two important selection biases: MSM recruited predominantly at a gay health centre with a focus on STI services, or at sexual venues, respectively, are likely to over-represent individuals at higher risk for acquiring STIs. However, because HCV-testing was primarily offered to MSM anonymously testing for HIV and/or syphilis, both HIV-positive MSM as well as those with past Syphilis infection are likely to be under-represented in the sample. Also, HIV-diagnosed MSM with known active or past HCV-co-infection were not included in our sample. Fourth, given the overall anti-HCV prevalence of 0.83% in our sample, this study is not sufficiently powered to rule out an odds ratio of less than 6.0 for the association between any potential risk factor and HCV seropositivity. However, our results on HCV prevalence among non-HIV-diagnosed MSM can probably be generalised to community-based samples of MSM in Switzerland. Finally, some distortions due to missing data and misclassifications cannot be excluded. In systematically screened MSM, urethral, pharyngeal, and rectal manifestations of gonorrhoea are about equally frequent [63,64], reflecting sexual practices of men who have sex with men. In contrast, in our sample, self-reported urethral gonorrhoea (19.6%) was more than ten times more common than pharyngeal (1.6%) or particularly rectal gonorrhoea (1.6%). This large discrepancy points towards a substantial under-diagnosis of rectal infections such as with *Gonococci* and *Chlamydia*, as suggested elsewhere [65]. We therefore believe that the real lifetime history of “lesion-prone STIs” is substantially higher than suggested by our proxy measure.

## Conclusions

Our findings suggest that in Zurich (Switzerland), prevalence of hepatitis C among MSM without diagnosed HIV is not higher than in the general population, where screening is not recommended. We found no evidence for elevated rates of sexual transmission of HCV among MSM without HIV diagnosis. Further studies are needed to clearly determine whether the identification of non-sexual exposures such as NIDU, tattoos or nosocomial exposures other than blood transfusion should prompt HCV-testing; however such approaches to increase the detection of hepatitis C should not only target MSM. Although trends in the spread of HCV among MSM should be closely monitored, for example in a national MSM cohort study, we currently see no reason for promoting routine HCV-testing for MSM without known HIV infection in Switzerland.

## Competing interests

AJS, LF, BZ, and AB have no competing interests to disclose. SR has served as an invited speaker for *Abbott.* BM has served as an advisor for *Merck, Abbott, Janssen, Gilead, Roche, Bristol-Myers Squibb* and *Boehringer* and has received grants from *Roche* and *Gilead.* PB has served as an advisor for *Merck*, *Abbott* and *Janssen* and has received grants from *Roche*, *Merck*, *Janssen*, *Gilead* and *Bristol-Myers Squibb.* No pharmaceutical company had any influence on the manuscript, the presented conclusions, or on any decision-making at *Checkpoint Zürich*.

## Authors’ contributions

AJS prepared the dataset, carried out the statistical analysis and wrote the manuscript. LF participated in the data preparation and the statistical analysis, provided administrative, technical, and logistic support, and largely contributed to the manuscript. BZ constructed the questionnaire and organised the fieldwork. SR supervised the laboratory analyses; AB and PB contributed to the manuscript; LF, BZ, BM, SR and PB designed the study; PB supervised the study and obtained funding. All authors read and approved the manuscript.

## Pre-publication history

The pre-publication history for this paper can be accessed here:

http://www.biomedcentral.com/1471-2458/14/3/prepub
